# Enhancing the Quality of Low-Alcohol Navel Orange Wine through Simultaneous Co-Fermentation Using *Saccharomyces cerevisiae* SC-125, Angel Yeast SY, and *Lactiplantibacillus plantarum* BC114

**DOI:** 10.3390/molecules29081781

**Published:** 2024-04-14

**Authors:** Hua Xiong, Yingyue Zhang, Wanting Wang, Hong Ye, Qing Zhang

**Affiliations:** 1 School of Food and Bioengineering, Xihua University, Chengdu 610039, China; xh87720551@163.com (H.X.); zhangyingyue20@163.com (Y.Z.);; 2 Food Microbiology Key Laboratory of Sichuan Province, Xihua University, Chengdu 610039, China; 3 Chongqing Key Laboratory of Speciality Food Co-Built by Sichuan and Chongqing, Xihua University, Chengdu 610039, China; 4 Sichuan Advanced Agricultural & Industrial Institute, China Agricultural University, Chengdu 611430, China

**Keywords:** navel orange fruit wine, *Saccharomyces cerevisiae*, *Lactiplantibacillus plantarum*, co-fermentation, volatile components

## Abstract

To date, there has been limited research on the interactive effects of yeast and lactic acid bacteria (LAB) on the sensory qualities of navel orange wine. In this study, using Jintang navel orange juice as the raw material, multi-microbial fermentation was conducted with *Saccharomyces cerevisiae* SC-125 and Angel yeast SY, as well as *Lactiplantibacillus plantarum* BC114. Single yeast and co-fermentation with *Lactiplantibacillus plantarum* were used as the control groups. The research aimed to investigate the physicochemical parameters of navel orange wine during fermentation. Additionally, headspace solid-phase microextraction gas chromatography–mass spectrometry (HP-SPME-GC-MS) was employed to determine and analyze the types and levels of flavor compounds in the navel orange wines produced through the different fermentation methods. The co-fermentation using the three strains significantly enhanced both the quantity and variety of volatile compounds in the navel orange wine, concomitant with heightened total phenol and flavonoid levels. Furthermore, a notable improvement was observed in the free radical scavenging activity. A sensory evaluation was carried out to analyze the differences among the various navel orange wines, shedding light on the impact of different wine yeasts and co-fermentation with LAB on the quality of navel orange wines.

## 1. Introduction

Navel oranges, belonging to the Rutaceae family and Citrus genus, have gained popularity among the various citrus varieties due to their unique flavor profile and relatively high phytochemical content [1]. In recent years, the cultivation of navel oranges has seen continuous expansion, with fruit growers advancing their cultivation techniques, resulting in a sustained increase in navel orange production [2]. The prolonged storage of navel oranges can lead to a decline in their quality, causing losses for growers. Therefore, there is a need for deep processing of navel oranges to extend their shelf life. Among the various processing methods, fermenting navel oranges into fruit wine is currently considered one of the more ideal approaches. Navel orange wine can effectively preserve a variety of the nutritional components present in navel orange pulp, such as organic acids, amino acids, polyphenols, and other bioactive compounds with antioxidant properties [3]. This not only minimizes the loss of nutritional value but also markedly contributes to filling gaps in the fruit wine market and expanding the range of fruit wine types available.

Currently, navel orange wine production mainly involves single-yeast fermentation or fermentation with non-*S. cerevisiae* strains [4]. Research efforts have primarily focused on optimizing the fermentation process of fruit wine, with limited attention given to the co-fermentation characteristics of yeast and lactic acid bacteria (LAB) in navel orange wines. Single-microorganism fermentation has clear application limitations [5], particularly in situations involving complex substrate compositions or intricate biochemical processes. In contrast, multi-strain co-fermentation, due to the complementary advantages among different strains, offers a fresh approach to overcoming the limitations of single-strain fermentation. For example, Favaretto et al. [6] conducted mixed fermentation of fruit residues using *S. cerevisiae* CAT-1 and *S. cerevisiae* Angel. Previous research [3] has demonstrated that co-fermentation using *S. cerevisiae* SC-125 and Angel yeast SY offers greater advantages compared to single-strain fermentation.

Studies have shown that the utilization of *Saccharomyces cerevisiae* in conjunction with *Lactiplantibacillus plantarum* during fruit wine co-fermentation notably enhances the abundance and diversity of aroma compounds in the resulting fruit wines [7]. In this regard, *Lactiplantibacillus plantarum* is employed in research to investigate its impact on the nutritional properties, volatile compounds, and sensory characteristics of fruit and vegetable juices, with the aim of enhancing the food’s attributes. Co-fermentation using *S. cerevisiae* and *L. plantarum* establishes a biologically mixed system, where microorganisms typically exhibit coordinated growth and metabolic interactions. This not only promotes their mutual growth but also yields certain compounds that are challenging to obtain in single-strain fermentations, thus imparting a distinctive flavor and mouthfeel to the wine [8,9]. For instance, Li et al. [10] conducted co-fermentation of apple cider using *S. cerevisiae* and *L. plantarum*. After 14 days, the concentration of glucuronic acid (GlcA) was measured to be 37.7 ± 1.7 mg/mL, which exceeded the levels achieved in the corresponding single-yeast fermentations.

With an improvement in living standards, low-alcohol fruit wines have gained increasing popularity. In order to enhance the flavor of navel orange wine and reduce its alcohol content, this study conducted a multi-microbial fermentation experiment by co-fermenting using *S. cerevisiae* SC-125, Angel yeast SY, and *L. plantarum* BC114 under optimal conditions. Various parameters during fermentation, such as live cell count, alcohol content, reducing sugars, organic acids, amino acids, total phenol, antioxidant properties, and changes in volatile aroma compounds in the navel orange wine, were investigated. The impact of yeast and LAB mixed fermentation on the quality and characteristics of the navel orange wine was further explored. These findings provide a theoretical basis for developing low-alcohol navel orange wines with a suitable taste and direction for the development of navel orange wines through co-fermentation using *S. cerevisiae* and *L. plantarum*.

## 2. Results and Discussion

### 2.1. Changes in Fundamental Parameters during the Fermentation Process

#### 2.1.1. Variations in Cell Count and pH during the Fermentation Process

**Figure 1:**
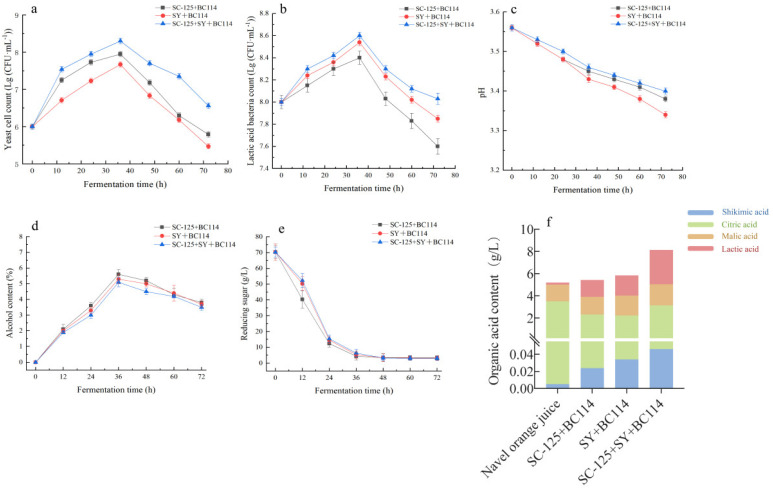
The changes in yeast cell count (**a**), lactic acid bacteria cell count (**b**), pH (**c**), alcohol content (**d**), and reducing sugars (**e**) during navel orange wine fermentation, and organic acids (**f**) in fermented navel orange wine.

In the early stages of navel orange fermentation, *S. cerevisiae* SC-125, Angel yeast SY, and *L. plantarum* BC114 exhibited robust metabolic capabilities, demonstrating excellent growth potential. As shown in Figure 1a and b, during the initial phase of co-fermentation in the sample group containing three strains, the cell counts of both the yeast and LAB rapidly increased to approximately 8.31 and 8.62 Log CFU/mL, respectively. This rapid growth can be attributed to the presence of reducing sugars in the navel orange juice, which provided essential nutrients for the yeast. As shown in Figure 1a, the biomass of all three groups exhibited an initial increase followed by a decrease. However, the quantity of *S. cerevisiae* SC-125 + Angel yeast SY + *L. plantarum* BC114 was conspicuously higher than the other two groups, and its deceleration trend was slower. *L. plantarum* BC114 utilized metabolic byproducts from the yeast and nutrients like reducing sugars to produce lactic acid and amino acids [11]. This resulted in an increase in the titratable acid content in the wine, leading to a decrease in pH (as shown in Figure 1c). The optimal pH range for fermented wines is, like wine, typically between 3.2 and 3.8. In all three different fermentation conditions for the navel orange wines, the pH remained within this range, with a decreasing trend. The pH level can significantly impact the content of higher alcohols in the final product, thereby influencing the flavor of the fruit wine.

#### 2.1.2. The Changes in Reducing Sugars and Alcohol Content during Fermentation

The utilization of reducing sugars reflects the yeast’s ability to carry out alcohol fermentation and substrate conversion. As shown in Figure 1d,e, during the initial phase of fermentation, the reducing sugar content decreased rapidly from 70.31 g/L in all three fermentation conditions, reaching 3.4 g/L in the *S. cerevisiae* SC-125 + *L. plantarum* BC114 group, 3.2 g/L in the Angel yeast SY + *L. plantarum* BC114 group, and 3.0 g/L in the *S. cerevisiae* SC-125 + Angel yeast SY + *L. plantarum* BC114 group. This met the standard for wine reducing sugars, which is below 4.0 g/L [12]. The alcohol content in the navel orange wines continued to increase and reached its peak at 36 h in all cases. During the period from 36 h to 72 h of fermentation, the alcohol content showed a decreasing trend in all three fermentation conditions. This may be due to *L. plantarum* BC114 utilizing reducing sugars for growth and reproduction through homolactic fermentation, which can contribute to the reduced alcohol content when co-fermented with *S. cerevisiae* SC-125 and Angel yeast SY. Throughout the entire fermentation process, the alcohol content remained around 0–6.0% during co-fermentation, which aligns with the low-alcohol wine standard of below 6.0%. The endpoint alcohol content for the co-fermentation group with the three strains was 5.2%.

#### 2.1.3. The Content of Organic Acids

Organic acids, as the most critical components responsible for the complex and diverse flavors in fruit, are closely related to juice acidity and diverse flavor characteristics. They also have a certain impact on the microbial community structure, fermentation rate, and shelf life of orange juice. To further investigate the changes in organic acids before and after navel orange fermentation, four organic acids (citric acid, malic acid, lactic acid, and shikimic acid) were analyzed. As shown in Figure 1f, among these, citric acid was the most abundant in the fresh navel orange juice, constituting approximately 80% of the total acid content. However, the citric acid content in all the fermented wines conspicuously decreased by 11.43% to 37.14% compared to the control (*p* < 0.05). Specifically, in the *S. cerevisiae* SC-125 + *L. plantarum* BC114 group, Angel yeast SY + *L. plantarum* BC114 group, and *S. cerevisiae* SC-125 + Angel yeast SY + *L. plantarum* BC114 group, the citric acid content decreased to 2.3 g/L, 2.2 g/L, and 3.1 g/L, respectively. In comparison to the fresh navel orange juice, the contents of malic acid, lactic acid, and shikimic acid in the navel orange wines produced using three fermentation methods were elevated to varying degrees. The lactic acid content showed a statistically significant increase, likely due to the substantial production of lactic acid by *L. plantarum* BC114. Similar studies [13] have also shown that *L. plantarum* can convert malic acid into lactic acid during fermentation, resulting in an increased lactic acid content. The oxalic acid content was relatively low in this study, with only 0.005 g/L detected in the fresh navel orange juice.

### 2.2. The Changes in Amino Acids before and after Fermentation

**Figure 2:**
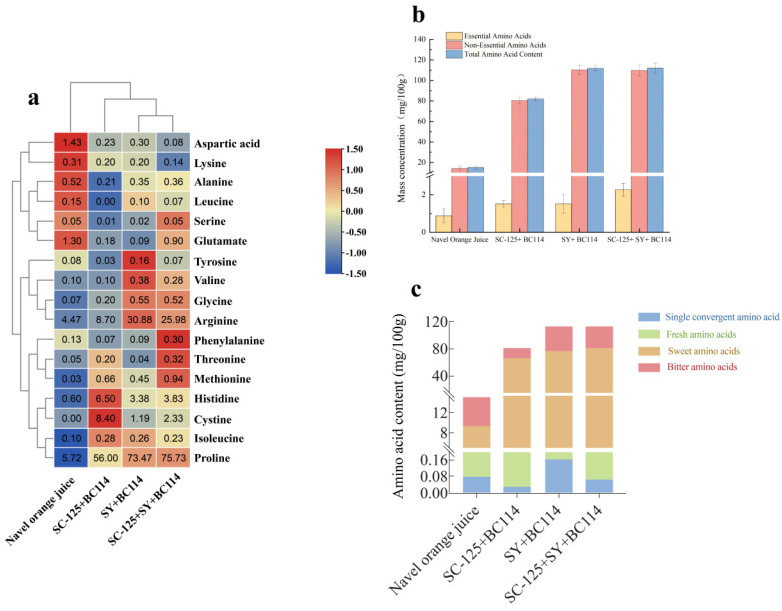
The composition and concentration of free amino acids in navel orange juice and fermented navel orange wines. (**a**) Changes in amino acid content in orange juice and navel orange wines produced by different fermentation methods (mg/100 g). (**b**) Composition of free amino acids in navel orange juice and fermented fruit wines (yellow: essential amino acids; red: non−essential amino acids; blue: total amino acids). (**c**) Classification of free amino acids in navel orange juice and fermented fruit wines (green: fresh amino acids; red: bitter amino acids; yellow: sweet amino acids; blue: single convergent amino acids).

Amino acids, the fundamental building blocks of proteins, also contribute to the unique flavor profiles of foods [14]. The varying levels of amino acids may influence the nutritional value and flavor of fruit wines. The changes in amino acids in the navel orange juice and navel orange wines produced using different fermentation methods are illustrated in Figure 2a. As shown in Figure 2b, after fermentation, the levels of essential amino acids, non-essential amino acids, and the total amino acid content in the navel orange wines increased. Among them, the group with the highest total amino acid content was the *S. cerevisiae* SC-125 + Angel yeast SY + *L. plantarum* BC114 group, which also had the highest content of essential amino acids. This indicates that the *S. cerevisiae* SC-125 + Angel yeast SY + *L. plantarum* BC114 group possesses higher nutritional value. The group with the highest non-essential amino acid content was the Angel yeast SY + *L. plantarum* BC114 group. As shown in Figure 2c, it can be observed that the predominant flavor-enhancing amino acids in navel oranges and their wines were sweet-tasting amino acids (serine, threonine, glycine, alanine, proline, valine, methionine, and cysteine), with the highest concentration in the *S. cerevisiae* SC-125 + Angel yeast SY + *L. plantarum* BC114 group, followed by the Angel yeast SY + *L. plantarum* BC114 group, the *S. cerevisiae* SC-125 + *L. plantarum* BC114 group, and finally the fresh navel orange juice. Furthermore, in comparison to previous experimental results [3], the sweet amino acid concentration in *L. plantarum* BC114, at 80.255 mg/100 g, surpasses that in the *S. cerevisiae* SC-125 + Angel yeast SY79 group, which stood at 79.515 mg/100 g. This suggests that the incorporation of *L. plantarum* BC114 can modestly heighten the sweetness of navel orange wines. Bitter-tasting amino acids (leucine, isoleucine, and valine) were less abundant than the sweet-tasting ones, and their concentration followed a similar pattern to the sweet-tasting amino acids. The fresh navel orange juice contained the highest amount of umami-tasting amino acids (aspartic acid and glutamic acid), followed by the *S. cerevisiae* SC-125 + Angel yeast SY + *L. plantarum* BC114 group.

### 2.3. The Content of Volatile Compounds

**Figure 3:**
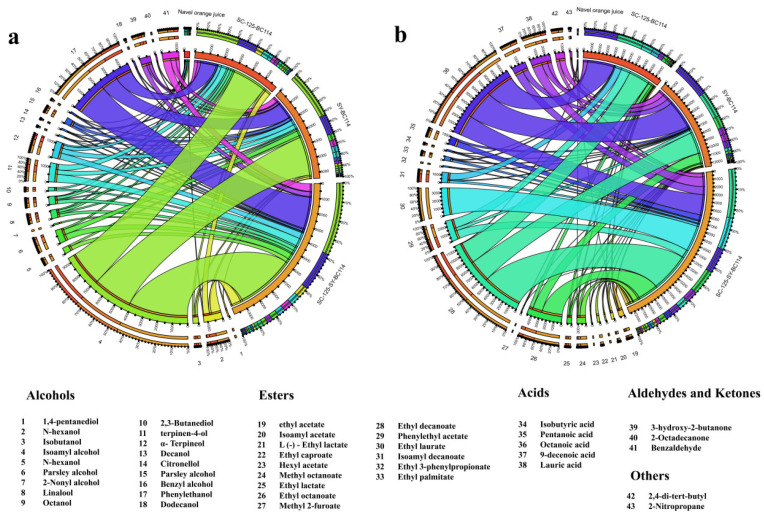
Distribution of 43 key volatile compounds in fermented navel orange fruit wine: (**a**) alcohols, acids; (**b**) esters, aldehydes and ketones, and other classes. The width of each bar in the compound’s histogram indicates its proportional distribution in the sample.

The changes in the types and content of volatile compounds in the navel orange juice and fermented navel orange wines are shown in Figure 3 and Table S2. A total of 43 volatile compounds were detected using HP-SPME-GC-MS. In the navel orange juice, seven alcohols, three esters, one acid, two aldehydes and ketones, and other compounds were identified. In the *S. cerevisiae* SC-125 + *L. plantarum* BC114 wine, there were 12 alcohols, 11 esters, 3 acids, 2 aldehydes and ketones, and 3 other compounds. In the SY + *L. plantarum* BC114 wine, there were 16 alcohols, 14 esters, 5 acids, 2 aldehydes and ketones, and 5 other compounds. In the *S. cerevisiae* SC-125 + Angel yeast SY + *L. plantarum* BC114 wine, there were 18 alcohols, 15 esters, 5 acids, 2 aldehydes and ketones, and 5 other compounds. The detected volatile compounds and their concentrations in the *S. cerevisiae* SC-125 + Angel yeast SY + *L. plantarum* BC114 group were the highest, followed by the Angel yeast SY + *L. plantarum* BC114 group, the *S. cerevisiae* SC-125 + *L. plantarum* BC114 group, and navel orange juice. Comparing with a previous co-fermentation experiment without *L. plantarum* BC114 [3], some key volatile compounds such as ethyl acetate, ethyl laurate, and ethyl caproate increased from 364.86 μg/L, 424.61 μg/L, and 135.25 μg/L to 373.72 μg/L, 3634.8 μg/L, 303.29 μg/L, respectively. This indicates that *L. plantarum* BC114 can significantly enhance the aromatic profile of navel orange wine, enhancing the fruity and sweet notes.

It is worth noting that when the concentration of alcohol compounds exceeds 400.0 mg/L, it may negatively impact the overall aroma of the wine and lead to a decrease in product quality [15]. In this study, the concentration of alcohol compounds ranged from approximately 332.34 to 10,016.49 ug/L, significantly lower than 400 mg/L. Among the three fermentation methods, the highest concentrations of alcohol compounds were observed for isobutanol and phenylethanol, with the *S. cerevisiae* SC-125 + Angel yeast SY + *L. plantarum* BC114 group having significantly higher levels than the other two fermentation groups. These alcohol compounds contribute to grainy, rose, and honey aromas in navel orange wines.

Ester compounds can impart sweetness and a fruity aroma to navel orange wine. In the navel orange juice, three ester compounds were detected, namely, ethyl acetate, methyl octanoate, and ethyl decanoate. Ethyl acetate, when present at levels below 150.0 mg/L, can enhance the taste and aroma of the wine [16]. After co-fermentation with yeast and LAB, the levels of ester compounds notably increased, contributing to the floral and fruity aromas in the navel orange wines. Similar results showing an increase in ester compounds with mixed fermentation were also reported by Hu et al. [17]. This may be attributed to the positive interactions occurring between the yeast and LAB. Such beneficial synergistic effects may also involve shared substrate metabolism and reciprocal influences on metabolite production, thereby impacting the generation and composition of volatile compounds in the navel orange wine. By exploring these synergistic effects, we can gain a better understanding of the collaborative relationship between yeast and lactic acid bacteria in navel orange wine fermentation. This insight is valuable for optimizing fermentation processes and enhancing the overall quality of navel orange wines.

Acidic compounds are indispensable contributors to the overarching flavor profile of wines, playing a pivotal role in shaping their sensory characteristics. However, excessive acidity can lead to a decrease in the overall flavor quality of the wine [18]. In this study, it was found that after mixed fermentation, the *S. cerevisiae* SC-125 + Angel yeast SY + *L. plantarum* BC114 group had the lowest hexanoic acid content, while the *S. cerevisiae* SC-125 + *L. plantarum* BC114 group had the highest content. When the content exceeds the aroma threshold, a rancid taste will emerge due to the pungent and sour flavors of caprylic acid [19]. Similarly, hexanoic acid can contribute a rancid flavor when present above its aroma threshold, due to its pungent and acidic characteristics [20]. Ketone compounds are known for their strong flavors, even at low concentrations, and can have a positive impact on various wines. The main volatile ketone identified after fermentation was 3-hydroxy-2-butanone, contributing a creamy aroma to navel orange wines. The concentration of this ketone significantly increased in all three fermentation methods, with the *S. cerevisiae* SC-125 + Angel yeast SY + *L. plantarum* BC114 group exhibiting the highest concentration. The concentrations and types of volatile compounds can greatly influence the aroma and flavor profile of navel orange wines, and the specific concentrations mentioned in this study can provide valuable insights for product development and quality control.

**Figure 4:**
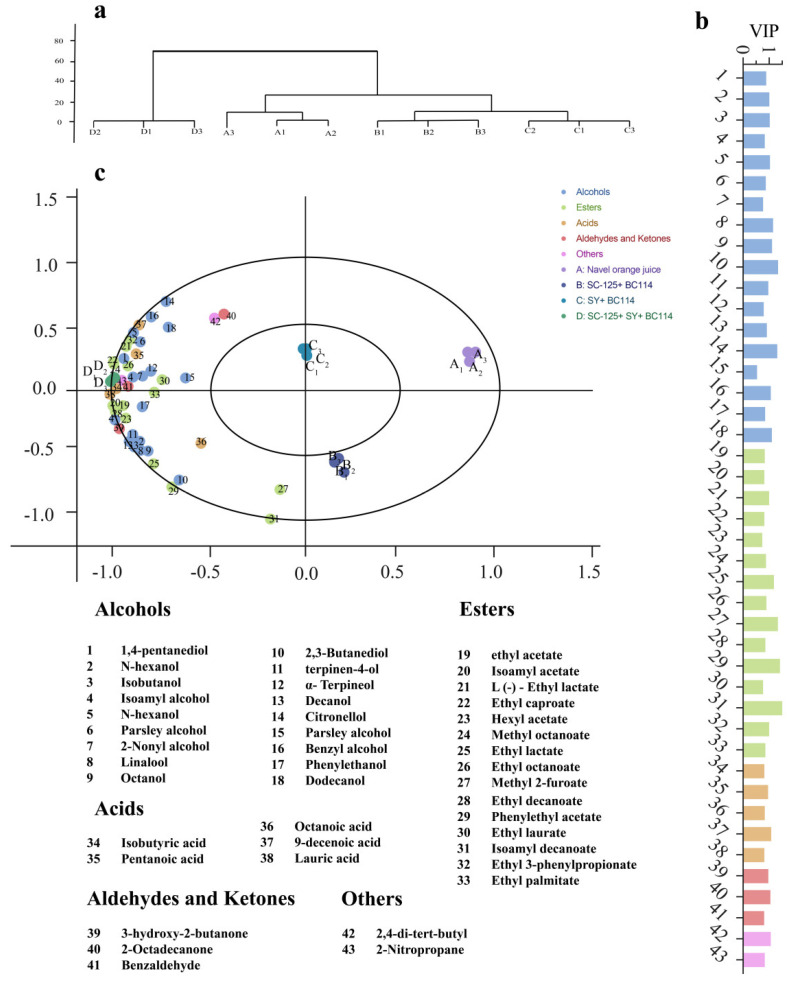
Analysis of volatile compounds in fermented navel orange wine samples using different fermentation methods. (**a**) Conventional clustering of volatile compounds using the average connection age method. (**b**) Important variables in predicting (VIP) values for 43 important volatile compounds. (**c**) The hyperbolic plot of volatile compounds in the orthogonal Partial Least Squares Discriminant Analysis (PLS–DA) model between different samples.

To provide a more intuitive analysis of the differences in aroma compounds obtained through various inoculation methods in navel orange wines, a Partial Least Squares Discriminant Analysis (PLS−DA) was conducted (Figure 4) using SIMCA 14.1 software. The PLS−DA model decomposes the information in the X-axis matrix into two categories: information related to Y (grouping) and unrelated information, effectively filtering out variables unrelated to grouping. Furthermore, by considering Variable Importance in Projection (VIP) scores, it enhances the reliability of discriminating differential metabolites. Figure 4c illustrates the correlation between different volatile compounds and the three groups of fermented samples. Compounds located farther from the coordinate center point indicate a greater contribution of that compound to the differences between samples. Differences in volatile compounds arise from the various fermentation conditions, which were particularly notable in the mixed fermentation with the three strains. The co-fermentation with the three strains resulted in a diverse and abundant array of volatile compounds, making a profound contribution to the distinctive flavor profile of the sample. The richness of these volatile compounds may add layers to the overall taste and aroma, enhancing the unique characteristics of the final flavor experience.

### 2.4. Changes in Total Phenol and Total Flavonoid Contents during the Fermentation Process

**Figure 5:**
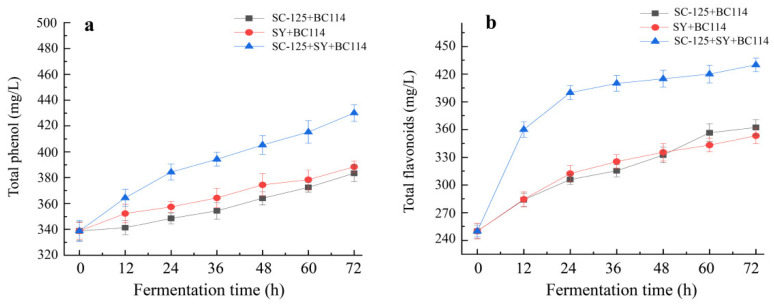
Changes in total phenol (**a**) and total flavonoid levels (**b**) during fermentation of navel orange fruit wine.

From Figure 5a,b, it can be observed that the total phenol and total flavonoid contents in the navel orange wines produced using the three fermentation methods increased with time. This increase in content is likely due to the action of glucosidases produced by *Lactobacillus plantarum*, which leads to the metabolism and release of phenolic substances, consequently resulting in an increase in both total phenol and total flavonoid levels. In the *S. cerevisiae* SC-125 + *L. plantarum* BC114 group and the Angel yeast SY + *L. plantarum* BC114 group, the total phenol content increased from 338.78 mg/L to 383.40 mg/L and 388.42 mg/L, respectively. Meanwhile, the total flavonoid content increased from 250.02 mg/L to 362.15 mg/L and 353.27 mg/L, respectively. It is worth noting that certain components of the flavonoid group may undergo degradation during the fermentation process. Notably, in the *S. cerevisiae* SC-125 + Angel yeast SY + *L. plantarum* BC114 group, the total phenol and total flavonoid contents increased conspicuously, reaching 430.21 mg/L and 420.11 mg/L, respectively. This represented a 26.5% and 72% increase, which not only surpass those of the other two groups but also exceed the results from a previous study using *S. cerevisiae* SC-125 + Angel yeast SY [3]. This indicates that co-fermentation with *S. cerevisiae* SC-125 + Angel yeast SY + *L. plantarum* BC114 has positive effects on enhancing phenolic and flavonoid substances.

### 2.5. Changes in Free Radical Scavenging Activity during the Fermentation Process

**Figure 6:**
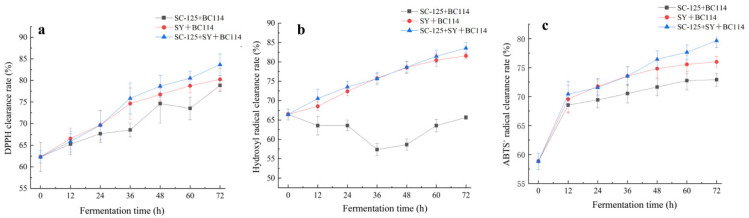
Changes in DPPH radical (**a**), hydroxyl radical (**b**), and ABTS^+^ (**c**) radical during fermentation of navel orange fruit wines.

Navel oranges are renowned for their abundance of flavonoids, vitamin C, limonene, carotenoids, and other compounds that endow them with potent antioxidant properties, promoting good health. To evaluate the antioxidant activity of the fermented navel orange wines, we examined the changes in DPPH free radical scavenging activity (Figure 6a). Following fermentation, all the fermented samples exhibited a statistically significant increase in DPPH levels, ranging from 78.85% to 83.65%. Notably, the sample co-fermented with the three strains displayed the highest DPPH level at 83.65%. This elevation in DPPH levels can be attributed to the notably improved utilization of antioxidant components such as phenols and flavonoids, which possess proton-donating properties, thanks to the fermentation process involving *L. plantarum* [21]. A study by Bai et al. [22] also noted that *L. plantarum* effectively enhanced the availability of compounds with proton-donating properties, leading to an increase in DPPH free radical scavenging activity.

The changes in hydroxyl free radicals during the fermentation process of the navel orange wines are depicted in Figure 6b. The samples fermented with *S. cerevisiae* SC-125 + Angel yeast SY + *L. plantarum* BC114 and Angel yeast SY + *L. plantarum* BC114 exhibited significant increases in hydroxyl free radicals. The hydroxyl free radical scavenging rates after fermentation rose to 83.54% and 81.54%, respectively, which represented an increase of 25.72% and 22.71% compared to pre-fermentation levels. After fermentation, the hydroxyl free radical scavenging rate in the *S. cerevisiae* SC-125 SY + *L. plantarum* BC114 sample was 64.55%, showing a 2.94% decrease compared to the control (66.45%). The degradation and oxidation of antioxidant compounds might be the reasons for the reduced hydroxyl free radical scavenging rate, as different strains of fermentation microorganisms have varying abilities to utilize antioxidant compounds [23]. Thus, the increase in hydroxyl free radical scavenging rate in navel orange wines appears to be closely related to the choice of strains during fermentation.

As indicated in Figure 6c, in all three fermentation group samples, the ABTS^+^ free radical scavenging activity in the navel orange wines increased significantly compared to the navel orange juice. Specifically, the samples fermented with *S. cerevisiae* SC-125 + Angel yeast SY + *L. plantarum* BC114 showed increases in ABTS^+^ free radical scavenging rates ranging from 35.39%. Among these, the SC-125 + SY + BC114 sample exhibited the highest ABTS^+^ free radical scavenging rate at 79.65%, followed by the *S. cerevisiae* SC-125 + *L. plantarum* BC114 sample at 75.99% and the Angel yeast SY + *L. plantarum* BC114 sample at 75.92%. The differences in ABTS^+^ free radical scavenging rates during the fermentation process may be related to the impact of *L. plantarum* on the antioxidant content of the juice during fermentation [24]. However, it is evident that overall, the ABTS^+^ free radical scavenging rates in the navel orange samples markedly increased after fermentation. This indicates that co-fermentation with yeast and LAB has a notably positive effect on the ABTS^+^ free radical scavenging activity in navel orange wines.

### 2.6. Sensory Evaluation Analysis

**Figure 7:**
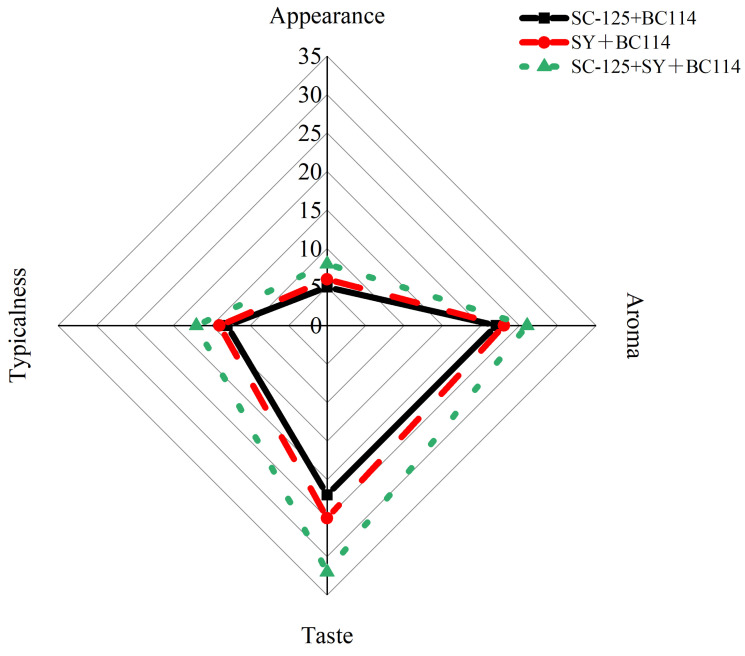
Sensory evaluation radar chart of navel orange wine samples from different fermentation groups.

Based on the radar chart in Figure 7, it is evident that there were statistically significant differences in the sensory evaluations of the navel orange wines produced by the different fermentation groups. The navel orange wines fermented with *S. cerevisiae* SC-125 + Angel yeast SY + *L. plantarum* BC114 exhibited higher scores in terms of appearance, aroma, taste, and typicality compared to the other groups. Additionally, the sensory evaluation scores of the SY + BC114 group were consistently higher than those of the *S. cerevisiae* SC-125 + *L. plantarum* BC114 group. In summary, the flavor of the navel orange wines from all three fermentation groups were perceived as pleasant and harmonious. Among them, the wines fermented with *S. cerevisiae* SC-125 + Angel yeast SY + *L. plantarum* BC114 exhibited the most favorable flavor, with a clearer body and distinct typical characteristics in terms of taste and appearance.

## 3. Materials and Methods

### 3.1. Strains and Materials

The commercial brewing yeast Angel yeast SY was obtained from Angel Yeast Co., Ltd. (Yichang, China). The *S. cerevisiae* SC-125 and *L. plantarum* BC114 strains were screened and preserved by the Sichuan Provincial Key Laboratory of Food and Biotechnology, College of Food and Bioengineering, Xihua University, Sichuan, China. In previous research, co-cultivating *S. cerevisiae* SC125 and *L. plantarum* BC114 resulted in an enhanced production of flavor compounds and GABA in a mulberry beverage [25]. Prior to inoculation, all strains were activated in potato glucose medium (200 g/L yeast extract, 20 g/L glucose, pH 7) for 48 h. Solid media were supplemented with agar (20 g/L).

The navel oranges were sourced from the Jintang navel orange orchard in Sichuan, China. Chemical reagents, including sodium hydroxide (Guangzhou CiShui Technology Co., Ltd., Guangzhou, China), 3,5-dinitrosalicylic acid (Chengdu Standard Substances Co., Ltd., Chengdu, China), glucose, sec-caprylic alcohol, potassium sodium tartrate (Chengdu Kelong Chemical Reagent Factory, Chengdu, China), anhydrous sodium nitrite (Shouguang Bangzehua Industrial Co., Ltd., Shouguang, China), Folin–Ciocalteu reagent, DPPH (2,2-diphenyl-1-picrylhydrazyl), phenol (Jinan Zesheng Chemical Co., Ltd., Jinan, China), sulfuric acid (Meishan Xinghongsheng Chemical Co., Ltd., Meishan, China), gallic acid, citric acid, pectinase, potassium metabisulfite, resorcinol (Shanghai Yuanye Bio-Technology Co., Ltd., Shanghai, China), and DNS (3,5-dinitrosalicylic acid) (Fucheng Chemical Reagent Co., Ltd., Tianjin, China) were of analytical grade. The navel orange juice and fermented navel orange wine were used as samples for analysis.

### 3.2. Determination of Colony Counts and pH during the Fermentation Process

The brewing process followed the methods outlined in Ref. [3]. After activating the *S. cerevisiae* SC-125, Angel yeast SY, and *L. plantarum* BC114 with a seed liquid concentration of 10^6^ CFU·mL^−1^, co-fermentation was conducted under the optimal conditions as follows: total inoculum 4%, fermentation temperature 30 ℃, fermentation time 72 h. During co-fermentation of the two strains, each group was inoculated with *L. plantarum* BC114 at a ratio of 1:1 (*v*/*v*) with *S. cerevisiae* SC-125 and Angel yeast SY. In the case of simultaneous fermentation with the three strains, the inoculation ratio of *S. cerevisiae* SC-125 to Angel yeast SY was 1:4 (*v*/*v*). Importantly, the combined ratio of these two strains was equivalent to that of *L. plantarum* BC114, both being 1:1 (*v*/*v*). Samples were taken every 12 h during this period. The supernatant was collected in a laminar flow hood and stored at −50 ℃. The colony counting method using PDA agar plates was employed to determine the cell counts of *S. cerevisiae* SC-125, Angel yeast SY, and *L. plantarum* BC114 in the mixed fermentation of navel orange wine at different fermentation stages. Additionally, separate fermentations with strain SC-125 and strain BC114, as well as strain SY and strain BC114, were conducted as controls. Cell counts were expressed as colony-forming units per milliliter (CFU·mL^−1^). The pH was measured using a pH meter (pHS-3C, Chengdu Ark Science and Technology Co., Ltd., Chengdu, China).

### 3.3. Determination of Reducing Sugars, Alcohol, Organic Acids, Amino Acids, and Volatile Compounds during the Fermentation Process

The determination of reducing sugars in the samples was performed following the method described by Zhang et al. [3]. Dilute sample 200 times with distilled water, mix with DNS reagent, incubate, cool, adjust to 25 mL, transfer to a 96-well plate, measure absorbance at 540 nm, and determine reducing sugar content from a standard curve regression equation. Alcohol content was determined using gas chromatography (QP2010PLUS, Shimadzu Corporation, Kyoto, Japan) using the external standard method [26]. The conditions were as follows: initial temperature 45 °C, 5 min hold, ramp to 60 °C at 3 °C/min, 3 min hold, further ramp to 200 °C at 20 °C/min, and 5 min hold; column: Rtx-Wax (30 m × 0.25 mm × 0.25 μm); carrier gas: high-purity helium at 1.0 mL/min; injection port temperature: 200 °C. The content of organic acids in the samples was determined following the method outlined by Gomis et al. [27]: column: ZORBAX SBAQ (250 mm × 4.6 mm, 5 μm, Agilent Technologies, Santa Clara, California, USA); detection: UV at 210 nm; temperature: 30 °C; flow: 0.8 mL/min; mobile phase: 97:3 KH_2_PO_4_ (0.01 M, pH = 2.62)–methanol; A: 0.01 M KH_2_PO_4_ (pH adjusted to 2.8 with H_3_PO_4_); B: 3% methanol; flow: 0.5 mL/min; injection volume: 10 μL; column temperature: 30 °C. The peak retention time and area of the samples were determined by comparing them with the organic acid standards and constructing a calibration curve.

The amino acid content in the samples was measured using a fully automated amino acid analyzer (L-8900, Hitachi Ltd., Kyoto, Japan) [28]. The samples were subjected to protein hydrolysis, obtaining a test solution after filtering through a 0.22 μm membrane. The amino acid analysis was performed using an automatic amino acid analyzer. The conditions were as follows: chromatography: C18 column; detection at 340 nm and 450 nm; column temperature: 40 °C; flow rate: 1.0 mL/min; mobile phase A: 20 mmol/L sodium acetate; mobile phase B: 20 mmol/L sodium acetate–methanol–acetonitrile (1:2:2). The analysis of volatile compounds was carried out using headspace solid-phase microextraction coupled with gas chromatography-mass spectrometry (HS-SPME/GC-MS) (GCMS2020NX, Shimadzu Corporation, Kyoto, Japan) [29]. The internal standard method was employed, using 0.4175 mg/L decanol as the internal standard. A 3.8 mL fermentation sample was mixed with 0.2 mL diluted sec-caprylic alcohol (diluted 1 × 10^5^ times with ultrapure water), sealed with 1 g NaCl, and equilibrated at 60 °C for 20 min. An SPME fiber was inserted, adsorbed for 20 min, withdrawn, and desorbed at 220 °C for 3 min in the gas chromatograph’s injection port.

### 3.4. Determination of Total Phenol and Total Flavonoid Contents and Antioxidant Activity during the Fermentation Process

The total phenolic content was determined using the Folin–Ciocalteu reagent (FCR) method with UV–visible spectrophotometry (WFJ7200 Unico Instruments, Shanghai) [30], employing rutin as a standard, while the total flavonoid content was measured using the aluminum chloride method [31]. The DPPH radical scavenging activity was determined according to the method outlined by Gulcin [32], hydroxyl radical scavenging activity was measured following the procedure by Tian [33], and ABTS^+^ radical scavenging activity was determined based on the method described by Lee [34]. For measuring DPPH scavenging activity, 1 mL of the navel orange beverage was mixed with 1 mL 0.5 mmol/L DPPH, incubated for 30 min, and A_1_ was measured at 517 nm. Water was used as a blank, and ethanol was used as a control. For measuring hydroxyl scavenging activity, ferrous sulfate, salicylic acid, wine, and H_2_O_2_ were combined. After 30 min at 37 °C, A_1_ was measured at 510 nm. Methanol was used as the blank, with water substituting for H_2_O_2_. For measuring ABTS^+^ scavenging activity, the sample was mixed with ABTS^+^ and the absorbance was measured at 734 nm. The clearance rate was calculated using the formula clearance rate (%) = [1 − (A_1_ − A_2_)/A_0_] × 100%, where A_0_ is the water absorbance and A_2_ is the ethanol absorbance.

### 3.5. Sensory Evaluation

After the completion of fermentation, a sensory evaluation of the navel orange fermented wine was conducted [35]. A panel of 10 trained assessors formed the judging team and assessed the navel orange wine based on the criteria listed in Table S1.

### 3.6. Statistical Analysis

Bar charts and line graphs were generated using GraphPad (version 9), and the principal component analysis (PCA) comparing aroma characteristics was performed using Origin 2018 and SIMCA 14.1. Heatmaps were constructed using TBtools-ll (Toolbox for Biologists) v1.116 software. The distribution of important volatile compounds was visualized using Circos software (http://circos.ca/, accessed on 1 February 2024).

## 4. Conclusions

Through the study of three different fermentation methods, it was discovered that using a combination of *S. cerevisiae* SC-125 + Angel yeast SY + *L. plantarum* BC114 for co-fermentation is the optimal approach for achieving the best sensory experience for navel orange wines. The collaborative fermentation of these three strains offers the production of a low-alcohol fermentation wine with distinctive qualities. Additionally, co-fermentation significantly enhanced the formation of essential amino acids and sweet amino acids. A total of 43 volatile compounds were detected in the fermented wine, primarily including alcohols, esters, acids, and ketones. Co-fermentation with these three strains not only increased the quantity and variety of volatile compounds but also elevated the total phenol and total flavonoid contents, achieving 430.21 mg/L and 420.11 mg/L, respectively. The in vitro antioxidant activity analysis indicated that the navel orange wine co-fermented with these strains exhibited higher scavenging rates for DPPH, hydroxyl, and ABTS^+^ free radicals, reaching 83.65%, 64.55%, and 64.55%, respectively. The sensory evaluation results demonstrated that the navel orange wine co-fermented with these strains displayed an intense color and a pleasant taste, garnering the highest sensory evaluation scores. These research findings provide an essential theoretical support for the development of navel orange wines utilizing brewing yeast and LAB, thereby enhancing the overall quality and sensory experience. Subsequent studies can perform further analysis at the molecular level, such as whole-genome or microbial community analysis, to gain deeper insights.

## Data Availability

No new data were created or analyzed in this study. Data sharing is not applicable to this article.

## Supplementary Materials

The following supporting information can be downloaded at: https://www.mdpi.com/article/10.3390/molecules29081781/s1.
