# Supplementary material for: Enhancing the Quality of Low-Alcohol Navel Orange Wine through Simultaneous Co-Fermentation Using Saccharomyces cerevisiae SC-125, Angel Yeast SY, and Lactiplantibacillus plantarum BC114
Source: Molecules. 2024 Apr 14;29(8):1781. doi: 10.3390/molecules29081781 (PMC11052192; doi:10.3390/molecules29081781)
Supplement: Supplementary file 1 [file molecules-29-01781-s001.zip › molecules-2915386-supplementary.pdf]

Table S1 Sensory evaluation of navel orange wine

Project	Scoring Criteria	Score (Points)
Appearance (Total Score: 10)	Clear and transparent, golden yellow, glossy	9~10
	Clear and transparent, golden yellow, with no visible suspended	7~8
	Clear, pale yellow, with a small amount of suspended particles	5~6
	Slightly cloudy, without gloss	0~4
Aroma (Total Score: 30)	Harmoniously balanced fruity and wine aromas with elegance	26~30
	Fruity and wine aromas are good, still enjoyable	21~25
	Limited fruit and wine aromas, with no off-flavors	11~20
	Insufficient fruit aroma, or there may be off-flavors	0~10
Flavor (Total Score: 40)	The wine is full-bodied, rich, pleasant, with subtle bitterness	30~40
	The wine is smooth, soft, and refreshing with balanced	21~30
	Balanced wine, pure without impurities, with a stronger bitter	11~20
	Thin and unbalanced wine, overly bitter and astringent	0~10
Typical (Total Score: 40)	Typically perfect, uniquely styled, flawlessly elegant	18~20
	Clearly typical, with a good style	15~17
	Clearly typical, with a good style	11~14
	Lacks typical characteristics, or has defects	0~10

Table S2 Main aroma components of navel orange beverage before and after fermentation

Category	Aroma components	Navel orange juice	1	2	3
1	1,4-pentanediol	0	0	16.66	79.14
2	N-hexanol	55.12	408.5	138.59	654.76
3	Isobutanol	0	125.77	104.49	141.39
4	Isoamyl alcohol	0	2443.68	2758.52	4143.61
5	N-hexanol	0	0	138.59	272.06
6	Parsley alcohol	0	117.99	363.89	414.86
7	2-Nonyl alcohol	0	0	0	94.06
8	Linalool	86.13	171.3	118.57	198.17
9	Octanol	42.91	187.91	76.45	251.05
10	2,3-Butanediol	0	117.77	0	124.12
11	Terpinen-4-ol	0	391.44	312.51	563.37
12	α- Terpineol	94.16	241.54	347.1	442.1
13	Decanol	0	30.92	6.48	64.34
14	Citronellol	18.67	0	31.84	53.1

15	Parsley alcohol	0	98.48	194.49	134.52
16	Benzyl alcohol	9.26	0	31.83	73.48
17	Phenylethanol	26.09	1129.78	1152.68	2254.67
18	Dodecanol	0	0	49.62	57.69
19	Ethyl acetate	23.87	157.91	125.48	373.72
20	Isoamyl acetate	0	80.159	45.72	224.15
21	L (-) - Ethyl lactate	0	0	53.34	120.63
22	Ethyl caproate	0	0	24.37	303.29
23	Hexyl acetate	0	12.57	0.62	99.51
24	Methyl octanoate	23.65	126.15	293.21	727.56
25	Ethyl lactate	0	234.24	140.71	256.76
26	Ethyl octanoate	0	486.78	1339.98	2798.72
27	Methyl 2-furoate	0	53.13	33.72	15.79
28	Ethyl decanoate	64.57	2975.05	3024.9	5498.81
29	Phenylethyl acetate	0	1246.29	399.57	1129.7
30	Ethyl laurate	0	0	0	3634.8
31	Isoamyl decanoate	0	911.77	130.3	317.63
32	Ethyl 3-phenylpropionate	0	0	50.54	117.66
33	Ethyl palmitate	0	134.65	97.17	308.51
34	Isobutyric acid	0	46.64	49.86	136.76
35	Pentanoic acid	0	0	265.48	764.86
36	Octanoic acid	22.01	3763.01	3982.57	2859.33
37	9-decenoic acid	0	0	764.86	1108.43
38	Lauric acid	0	546.53	783.62	1347.66
39	3-hydroxy-2-butanone	24.65	212.75	152.56	327.04
40	2-Octadecanone	0	0	56.22	29.38
41	Benzaldehyde	7.78	226.21	356.54	537.86
42	2,4-di-tert-butyl	0	0	772.99	445.07
43	2-Nitropropane	0	42.36	65.44	154.65

Annotation: 1 represents SC-125 + BC114, 2 represents SY + BC114, 3 represents SC-125 + SY + BC114.

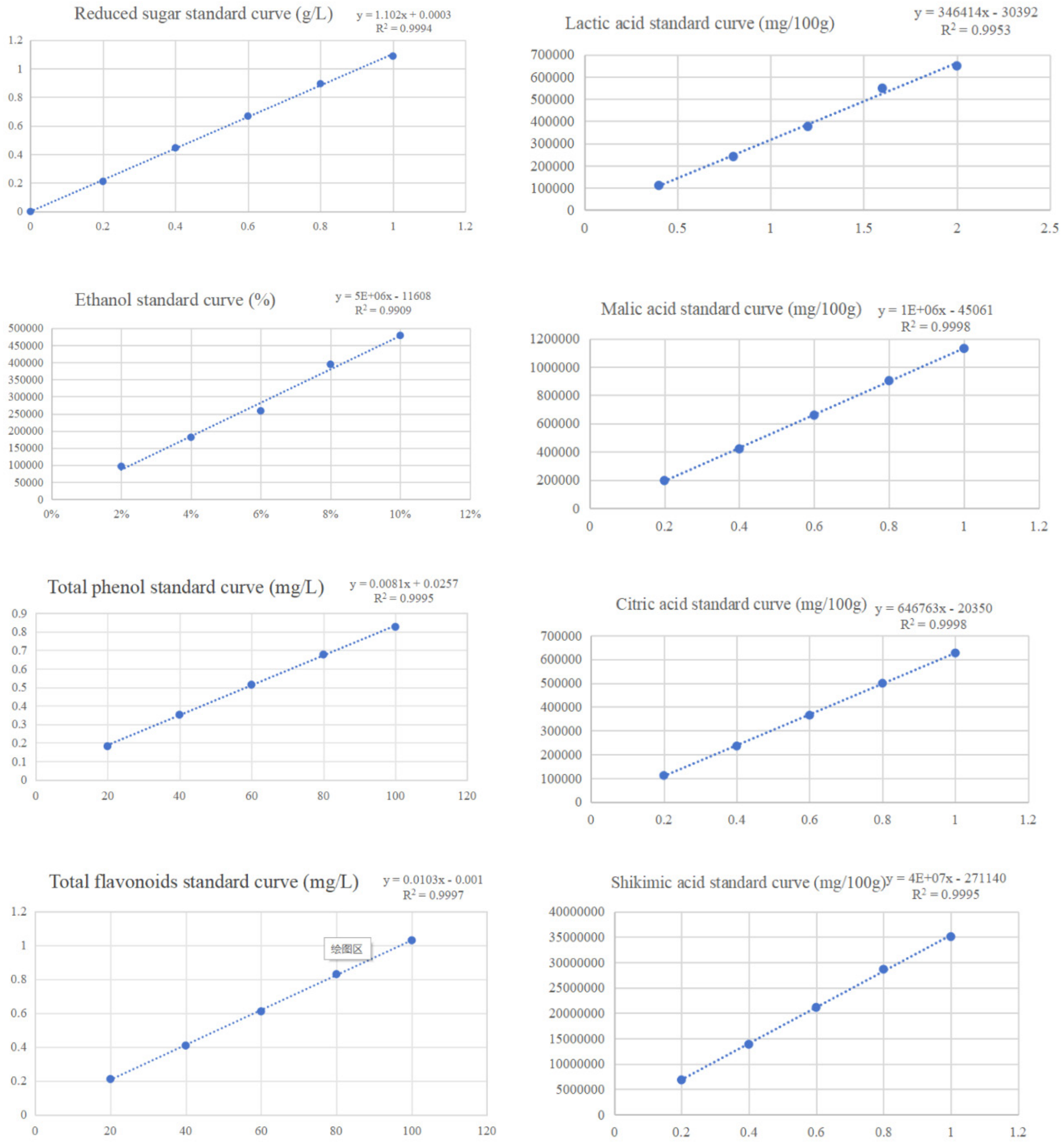

Figure S1 Standard curve
